# Avoidance of thiazoline compound depends on multiple sensory pathways mediated by TrpA1 and ORs in *Drosophila*

**DOI:** 10.3389/fnmol.2023.1249715

**Published:** 2023-12-22

**Authors:** Shoma Sato, Aliyu Mudassir Magaji, Makoto Tominaga, Takaaki Sokabe

**Affiliations:** ^1^Thermal Biology Group, Exploratory Research Center on Life and Living Systems, National Institutes of Natural Sciences, Okazaki, Japan; ^2^Division of Cell Signaling, National Institute for Physiological Sciences, National Institutes of Natural Sciences, Okazaki, Japan; ^3^Graduate Institute for Advanced Studies, SOKENDAI, Hayama, Japan

**Keywords:** repellent, TRP channel, TRPA1, odorant receptor, thiazoline compounds, sensory pathways, *Drosophila melanogaster*, calcium imaging

## Abstract

Transient receptor potential (TRP) channels are primary sensory molecules in animals and are involved in detecting a diverse range of physical and chemical cues in the environments. Considering the crucial role of TRPA1 channels in nocifensive behaviors and aversive responses across various insect species, activators of TRPA1 are promising candidates for insect pest control. In this study, we demonstrate that 2-methylthiazoline (2MT), an artificial volatile thiazoline compound originally identified as a stimulant for mouse TRPA1, can be utilized as a novel repellent for fruit flies, *Drosophila melanogaster*. We observed that 2MT induced strong, dose-dependent avoidance behaviors in adult males, regardless of their feeding states, as well as egg laying behavior in females. These aversive responses were mediated by contact chemosensation via TrpA1 and olfaction via odorant receptors. Knocking down *TrpA1* revealed the essential roles of bitter taste neurons and nociceptive neurons in the legs and labellum. Furthermore, among five isoforms, TrpA1-C and TrpA1-D exclusively contributed to the aversiveness of 2MT. We also discovered that these isoforms were directly activated by 2MT through covalent modification of evolutionarily conserved cysteine residues. In conclusion, we have identified 2MT as a stimulant for multiple sensory pathways, triggering aversive behaviors in fruit flies. We propose that 2MT and related chemicals may serve as potential resources for developing novel insect repellents.

## Introduction

Crop damage in agriculture and the transmission of vector-borne diseases by insect pests have become worldwide threat nowadays. Insecticides and repellents have been used as chemical treatments against insect pests for centuries (Debboun and Moore, 2006; Umetsu and Shirai, 2020). While insecticides are effective in controlling insect pest population, the majority of them have the potential to disrupt ecosystems, pose risks to human health, and contribute to the emergence of insecticide-resistant pests. Introducing insect repellents offers an alternative countermeasure to overcome these issues, and DEET (*N*,*N*-Diethyl-*meta*-toluamide) has been predominantly used since the 20th century (Leal, 2014; DeGennaro, 2015). However, due to limitations in its use and effectiveness, there is a high demand for the development of novel repellents. To discover compounds that effectively repel insect pests, it is important to focus on key molecules associated with sensory, particularly aversive, responses.

Insects detect volatile chemicals through odorant receptors (ORs), ionotropic receptors (IRs), gustatory receptors (GRs), and transient receptor potential (TRP) channels (Montell, 2021). Insects also detect non-volatile chemicals through IRs, GRs, TRP channels, Pickpocket (Ppk) channels, and Opsins (Montell, 2021). Among them, TRP channels play a key role in nocifensive behavior and aversive responses to various chemical stimuli in many insect species. For example, TRPC channels are activated by carbon dioxide, and the natural repellents, camphor, citronellal, and citronellol in the fruit fly, *Drosophila melanogaster* (Badsha et al., 2012; Zhang et al., 2013; Tian et al., 2022). A TRPM channel is activated by menthol in the red flour beetle, *Tribolium castaneum* (Shimomura et al., 2021). Hymenoptera-specific TRPA (HsTRPA) channel in the red imported fire ant, *Solenopsis invicta* is activated by citronellal, caryophyllene, octanoic acid, and decanoic acid (Wang X. et al., 2018). In the honey bee, *Apis mellifera*, HsTRPA is activated by allyl isothiocyanate (AITC), cinnamaldehyde, and camphor (Kohno et al., 2010). TRPA1 channels have been extensively studied across species and are activated by various natural irritants, such as citronellal, cinnamaldehyde, AITC, aristolochic acid, menthol, cinnamodial, and nepetalactone. These chemicals induce avoidance behaviors in *Drosophila melanogaster*, *Tribolium castaneum*, the cotton bollworm (*Helicoverpa armigera*) and multiple mosquito species (*Aedes aegypti*, *Anopheles gambiae*, *Anopheles stephensi*, and *Culex pipiens pallens*) (Kang et al., 2010; Kim et al., 2010; Kwon et al., 2010; Du et al., 2015; Wei et al., 2015; Inocente et al., 2018; Li et al., 2019; Boonen et al., 2021; Melo et al., 2021; Shimomura et al., 2022). Therefore, insect TRPA1 activators are promising leads for novel repellents with a broad spectrum.

Trimethylthiazoline (TMT), a volatile component of fox urine and feces, is known to elicit innate fear responses in rodents (Rosen et al., 2015). An artificial, volatile thiazoline compound, 2-methylthiazoline (2MT), can induce stronger fear responses than TMT in mice (Isosaka et al., 2015). 2-*sec*-butyl-4,5-dihydrothiazole (SBT), a volatile mouse alarm pheromone, also has a thiazoline structure similar to TMT and induces fear responses in mice (Brechbuhl et al., 2013). Physiological responses induced by these thiazoline-related compounds (TMT, SBT, and 2MT) and other artificial thiazoline analogs (Matsuo et al., 2021a) are mediated by TRPA1 (Wang Y. et al., 2018). TMT and 2MT are electrophilic chemicals that activate mouse TRPA1 by directly binding to cysteine residues in the N-terminal cytosolic domain (Wang Y. et al., 2018). This mechanism of TRPA1 activation by the covalent modification of cysteines is evolutionarily conserved across species (Kang et al., 2010). Notably, several of the corresponding cysteines react with 2MT in mouse TRPA1 are conserved across species, including insect pests (Kang et al., 2010; Kwon et al., 2010; Wei et al., 2015; Wang X. D. et al., 2021). Furthermore, 2MT effectively deters mice even without direct contact, likely due to its volatility (Wang Y. et al., 2018). These facts led us to investigate the efficacy of thiazoline-related compounds as insect repellents and their molecular targets in *Drosophila melanogaster*. Here, we found that 2MT induces strong aversive responses in *Drosophila* through multiple sensory pathways. This requires specific TrpA1 isoforms expressed in taste and nociceptive neurons, and 2MT directly activates these TrpA1 isoforms by biding to evolutionarily conserved cysteines. Additionally, our data reveals that olfactory sensory neurons (OSNs) expressing ORs can also recognize 2MT, particularly at low concentrations, enabling insects to escape from 2MT in a wide range of concentration. Based on these findings, we propose that 2MT functions as a novel insect repellent that stimulates multiple sensory pathways.

## Results

### *Drosophila* displayed avoidance responses to 2MT

To assess flies’ avoidance behavior toward chemicals, we employed a two-choice positional preference assay (Supplementary Figure 1A; Sanchez-Alcaniz et al., 2017). Starved adult males were released onto an assay plate coated with agarose containing sucrose. The plate was divided into four sections, with two diagonal quadrants containing a test chemical and the remaining two quadrants without the chemical. A group of approximately 50 flies was allowed to explore and select a location using their chemosensory abilities. Thus, the number of flies in each quadrant at a given time could reflect their decision, either preference or avoidance of the test chemicals. Although the preference index (PI) exhibited considerable fluctuations every minute throughout the experimental period (Supplementary Figure 1B), we observed consistent trends of PI decrease after calculating the moving averages over a 21-min interval (see Methods).

**FIGURE 1:**
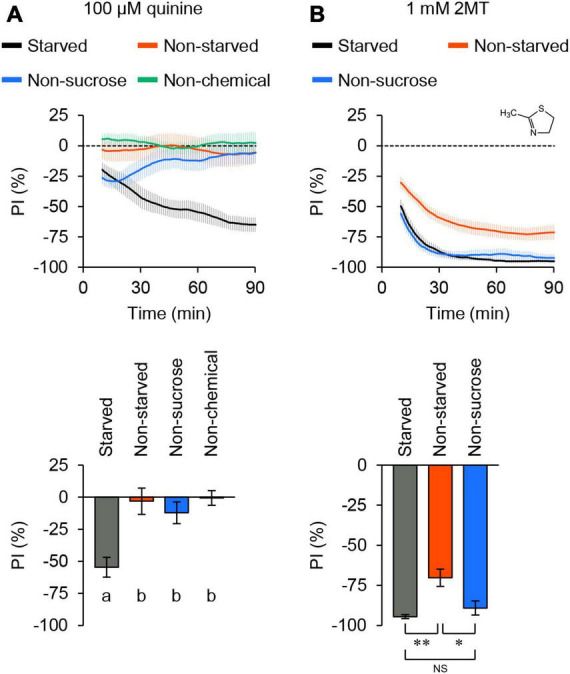
2MT evoked avoidance behavior in adult flies. The temporal changes of preference index (PI) to chemical stimulus (top) and the PI at 60 min (bottom) in control flies (*w*^1118^) over time. A dotted line in the top panel represents the level indicating no preference or avoidance. The data are presented as moving average ± SEM. **(A)** The PI of 24-hour-starved (black) or non-starved (red) flies for 100 μM quinine. PI of 24-hour-starved flies for 100 μM quinine in the absence of sucrose (blue). PI of 24-hour-starved flies in the absence of quinine (green). The same letters indicate no significant difference based on one-way ANOVA with Tukey’s multiple comparison (*N* = 8). **(B)** The PI of 24-hour-starved (black) or non-starved (red) flies for 1 mM 2MT. PI of 24-hour-starved flies for 1 mM 2MT in the absence of sucrose (blue). NS, not significant; *P < 0.05; **P < 0.01 by Kruskal-Wallis test with Steel-Dwass multiple comparison (*N* = 9).

When we tested quinine, known to elicit aversive responses in flies through bitter taste neurons (Wang et al., 2004), the flies displayed an increasing aversion to quinine over time (Figure 1A; Supplementary Figure 2A). In contrast, when quinine was absent from the agarose, flies distributed evenly across all quadrants. This result indicated that the assay could detect chemical avoidance in a population of flies. In contrast to starved flies, satiated flies did not exhibit avoidance of the same concentration of quinine (Figure 1A; Supplementary Figure 2A). Moreover, flies did not develop an aversion to quinine when sucrose was excluded from agarose (Figure 1A; Supplementary Figure 2A). These results suggested that quinine avoidance detected by this positional choice assay was primarily dependent on feeding behavior.

Next, we investigated 2MT, a volatile compound that induces innate fear responses in mice (Isosaka et al., 2015), and its derivatives are present in some fruits and vegetables (Servili et al., 2000; Fernandez et al., 2001; Takahashi and Shibamoto, 2008). Flies immediately and consistently displayed avoidance of 1 mM 2MT (Figure 1B; Supplementary Figure 2B). Unlike quinine, the avoidance of 2MT was observed regardless of the inclusion of sucrose in the agarose, and it was also evident in satiated flies (Figure 1B; Supplementary Figure 2B). In subsequent experiments, we conducted the positional choice assay using 24-hour-starved flies in the presence of 2 mM sucrose. Collectively, these findings suggest that 2MT avoidance is regulated not only by taste but also by other sensory pathways.

### TrpA1 and ORs play crucial role in mediating the avoidance of 2MT

**FIGURE 2:**
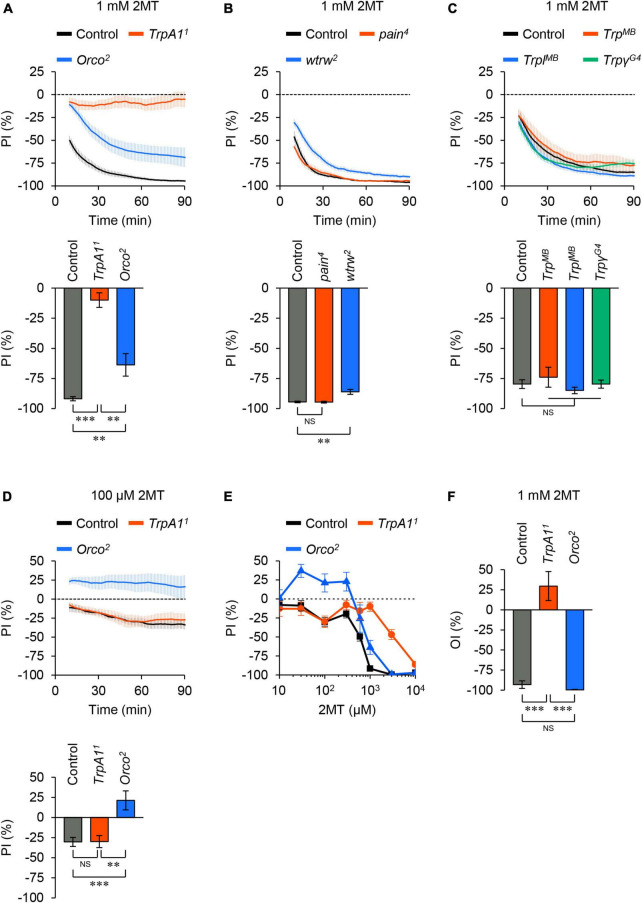
2MT avoidance was reduced in *TrpA1* and *Orco* mutants. **(A–C)** The temporal changes of PI to 1 mM 2MT (top) and the PI at 60 min (bottom) over time. A dotted line in the top panel represents the level indicating no preference or avoidance. The data are presented as moving average ± SEM. **(A)** The PI for 1 mM 2MT in control (*w*^1118^; black), *TrpA1^1^* (red), and *Orco*^2^ (blue) flies. **P < 0.01; ***P < 0.001 by Kruskal-Wallis test with Steel-Dwass multiple comparison (*N* = 9–12). **(B)** The PI for 1 mM 2MT in control (*w*^1118^; black), *pain*^4^ (red), *wtrw*^2^ (blue) flies. NS, not significant; **P < 0.01 by Kruskal-Wallis test with Steel multiple comparison (*N* = 8). **(C)** The PI for 1 mM 2MT in control (*w*^1118^; black), *Trp^MB03672^* (*Trp^MB^*; red), *Trpl^MB10553^* (*Trpl^MB^*; blue), *Trpγ^G4^* (green) flies. NS, not significant by Kruskal-Wallis test with Steel multiple comparison (*N* = 7–8). **(D)** The PI for 100 μM 2MT in control (*w*^1118^; black), *TrpA1^1^* (red), and *Orco*^2^ (blue) flies. NS, not significant; **P < 0.01; ***P < 0.001 by one-way ANOVA with Tukey’s multiple comparison (*N* = 9). **(E)** The dose dependency of PI for 2MT (10 μM–10 mM) at 60 min in control (*w*^1118^; black), *TrpA1^1^* (red), and *Orco*^2^ (blue) flies (*N* = 6–17). **(F)** The oviposition index (OI) for 1 mM 2MT in control (*w*^1118^; black), *TrpA1^1^* (red), and *Orco*^2^ (blue) flies. The data are presented as mean ± SEM. NS, not significant; ***P < 0.001 by Kruskal-Wallis test with Steel-Dwass multiple comparison (*N* = 11–12).

To determine which sensory molecules are involved in 2MT avoidance, we examined mutants of TRP channels known to be associated with chemical avoidance in adult flies (Al-Anzi et al., 2006; Kang et al., 2010; Kim et al., 2010; Kwon et al., 2010; Badsha et al., 2012; Zhang et al., 2013; Mandel et al., 2018; Li et al., 2020; Boonen et al., 2021; Melo et al., 2021). Null mutation of *TrpA1* nearly abolished avoidance responses to 1 mM 2MT (Figure 2A; Supplementary Figure 3A). In contrast, other TRP channel mutants, including TRPA subfamily (*painless*; *pain* and *water witch*; *wtrw*) and TRPC subfamily (*Trp*, *Trp-like*; *Trpl*, and *Trp*γ) showed minimal or no reduction in avoidance behavior to the same concentration of 2MT (Figures 2B, C; Supplementary Figures 3B, C). These results demonstrate the essential role of TrpA1 in detecting 2MT in flies.

Next, as 2MT is a volatile compound, we investigated whether olfaction was necessary for 2MT avoidance. We observed that flies carrying a null mutation of *Odorant receptor co-receptor* (*Orco*), resulting in olfactory dysfunction (Larsson et al., 2004), exhibited a partial but significant impairment in the avoidance response to 1 mM 2MT at all tested time points (30, 60, and 90 min) compared to the control (Figure 2A; Supplementary Figure 3A). *Orco* mutant flies did not avoid low concentrations of 2MT (30, 100, and 300 μM). Notably, *TrpA1* mutant flies displayed avoidance behavior indistinguishable from control flies at these concentrations (Figures 2D, E; Supplementary Figures 3D, 4). Therefore, we conclude that TrpA1 and ORs are involved in the contact chemosensory- and olfactory-dependent detection of 2MT, respectively, with their contributions depending on the concentration of the chemical.

### 2MT affects reproductive behavior in females through TrpA1

We investigated the impact of 2MT on another behavioral response, oviposition behavior, in females. Female flies use their chemosensory abilities to avoid laying eggs in the presence of harmful or aversive chemicals (Stensmyr et al., 2012; Melo et al., 2021). We conducted an oviposition assay similar to the two-choice positional preference assay by releasing adult females onto an agarose-coated plate divided into four sections. After 6 h, we counted the number of eggs in each quadrant and calculated the oviposition index (OI). Control and *Orco* mutant flies exhibited strong avoidance of laying eggs on agarose containing 1 mM 2MT (Figure 2F). In contrast, *TrpA1* mutant flies did not show this pronounced avoidance response (Figure 2F). These results indicate that 2MT inhibits egg laying in female flies via TrpA1. Thus, the action of 2MT through TrpA1 elicits general aversive responses in adult flies, as observed in both male flies (two-choice positional preference assay) and female flies (oviposition assay).

In mice, exposure to 2MT leads to freezing behavior and suppression of locomotor activity (Isosaka et al., 2015; Matsuo et al., 2021b). To assess the locomotor activities of flies during 2MT exposure, we recorded their spontaneous walking and measured the total distance moved during 10-min test intervals using a video-tracking system. Flies were exposed to 100 μM to 10 mM 2MT, and their locomotor activity was compared to that of flies not exposed to the chemical. In control flies, there were no differences in locomotor activity when exposed to any concentration of 2MT during all test intervals (Supplementary Figure 5A). *TrpA1* mutant flies appeared to exhibit increased movement compared to control flies in the absence or presence of 2MT up to 1 mM. However, their locomotor activity was reduced in the presence of 10 mM 2MT for unknown reasons (Supplementary Figure 5B). These results highlight the difference in the physiological effects of 2MT between mice and flies.

### A thiazoline-related compound 4E2MT induces aversive responses via TrpA1

**FIGURE 3:**
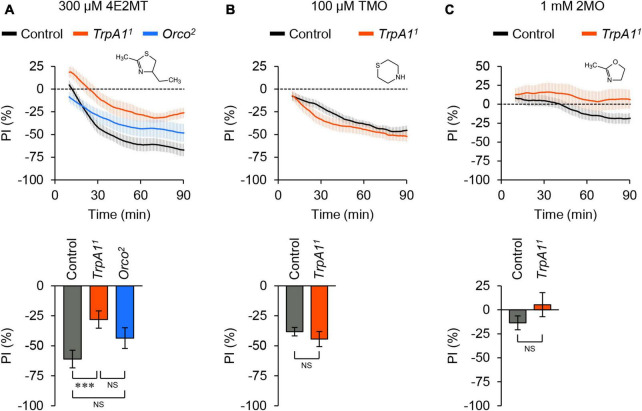
Thiazoline-related compounds evoked different behavioral responses. The temporal changes of PI to thiazoline-related compounds (top) and PI at 60 min (bottom). A dotted line in the top panel represents the level indicating no preference or avoidance. The data are presented as moving average ± SEM. **(A)** The PI to 300 μM 4E2MT in control (*w*^1118^; black), *TrpA1^1^* (red), and *Orco*^2^ (blue) flies. NS, not significant; ***P < 0.001 by one-way ANOVA with Tukey’s multiple comparison (*N* = 9). **(B)** The PI to 100 μM TMO in control (*w*^1118^; black) and *TrpA1^1^* (red) flies. NS, not significant by Student’s *t*-test (*N* = 10). **(C)** The PI to 1 mM 2MO in control (*w*^1118^; black) and *TrpA1^1^* (red) flies. NS, not significant by Student’s *t*-test (*N* = 8).

We also investigated the aversive responses induced by other thiazoline-related compounds, considering that some of these compounds can activate mouse TRPA1 (Matsuo et al., 2021a). 2-Methyl-4-ethylthiazoline (4E2MT) elicited avoidance behavior in control flies, which was partially reduced in *TrpA1* mutant flies (Figure 3A; Supplementary Figure 6A). Unlike 2MT, the avoidance response to 4E2MT was not affected in *Orco* mutant flies, although the chemical is volatile. Thiomorpholine (TMO) induced avoidance behavior in both control and *TrpA1* mutant flies (Figure 3B; Supplementary Figure 6B), suggesting that it acts on unknown molecules. 2-Methyl-2-oxazoline (2MO), which does not activate mouse TRPA1 (Wang Y. et al., 2018; Matsuo et al., 2021a), did not elicit clear behavioral responses in either control or *TrpA1* mutant flies (Figure 3C; Supplementary Figure 6C). In summary, among the tested thiazoline-related compounds, 2MT and 4E2MT exerted their aversive effects primarily through TrpA1, whereas only 2MT induced avoidance behavior through olfaction.

### Multiple sensory neurons are involved in 2MT avoidance

**FIGURE 4:**
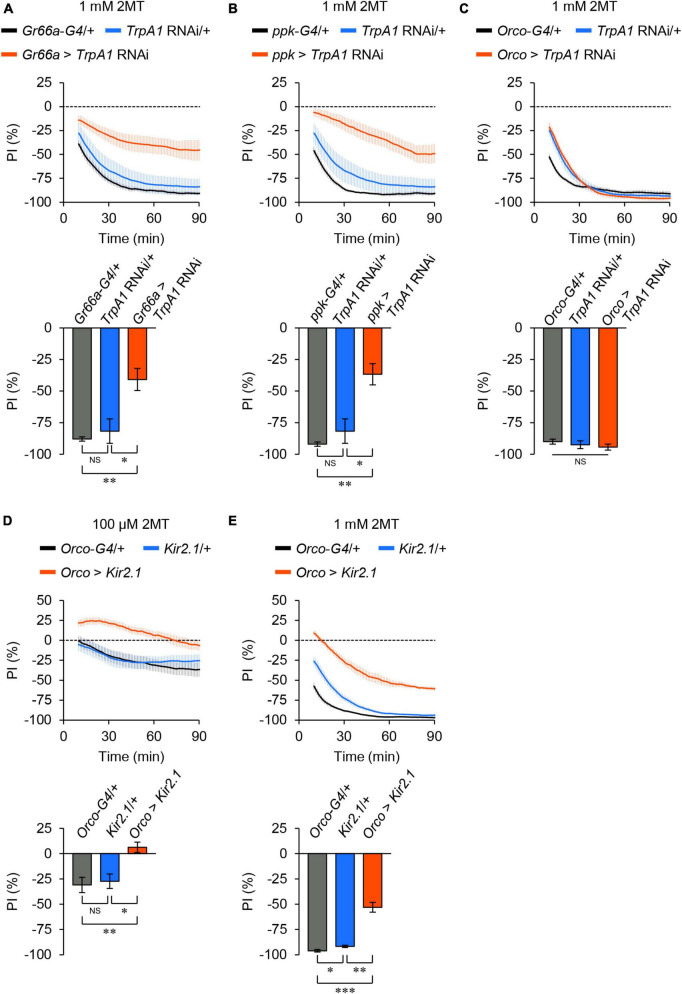
TrpA1 expression in taste and nociceptive sensory neurons was required for 2MT avoidance. The temporal changes of PI to 2MT (top) and the PI at 60 min (bottom). A dotted line in the top panel represents the level indicating no preference or avoidance. The data are presented as moving average ± SEM. **(A)** PI to 1 mM 2MT in *Gr66a-GAL4*/+ (*Gr66a-G4*/+; black), +*/UAS*-*dicer2*; +*/UAS*-*TrpA1* RNAi (*TrpA1* RNAi/+; blue), and *Gr66a-GAL4*/*UAS*-*dicer2*; +*/UAS*-*TrpA1* RNAi (*Gr66a* > *TrpA1* RNAi; red) flies. **(B)** The PI to 1 mM 2MT in *ppk-GAL4*/+ (*ppk-G4*/+; black), *TrpA1* RNAi/+ (blue), and +/*UAS*-*dicer2*; *ppk-GAL4*/*UAS*-*TrpA1* RNAi (*ppk* > *TrpA1* RNAi; red) flies. **(C)** The PI for 1 mM 2MT in *Orco-GAL4*/+ (*Orco-G4*/+; black), *TrpA1* RNAi/+ (blue), and *Orco-GAL4*/*UAS*-*dicer2*; +/*UAS*-*TrpA1* RNAi (*Orco* > *TrpA1* RNAi; red) flies. NS, not significant; *P < 0.05; **P < 0.01 by Kruskal Wallis test with Steel-Dwass multiple comparison [*N* = 8; note that *TrpA1* RNAi/+ data are shared between panels **(A,B)**]. **(D,E)** The PI to 100 μM 2MT **(D)** and 1 mM 2MT **(E)** in *Orco*-*GAL4*/+ (*Orco-G4*/+; black), +/*UAS*- *Kir2.1::GFP* (*Kir2.1*/+; blue), and *Orco*-*GAL4*/*UAS*-*Kir2.1::GFP* (*Orco* > *Kir2.1*; red) flies. NS, not significant; *P < 0.05; **P < 0.01; ***P < 0.001 by Kruskal-Wallis test with Steel-Dwass multiple comparison (*N* = 9–10).

*TrpA1* is expressed in bitter taste neurons (Kang et al., 2010, 2011; Kim et al., 2010; Leung et al., 2020), OSNs (Kwon et al., 2010), and nociceptive sensory neurons (Khuong et al., 2019) in adult flies. To determine which sensory neurons are involved in 2MT avoidance, we performed knockdown experiments targeting *TrpA1* in specific sensory neurons using the GAL4/UAS binary expression system and RNA interference (RNAi). Knockdown of *TrpA1* in bitter taste neurons using *Gr66a*-*GAL4* or in nociceptive neurons using *ppk*-*GAL4* significantly reduced 2MT avoidance (Figures 4A, B; Supplementary Figure 7A), whereas knockdown in the OSNs using *Orco*-*GAL4* did not have a significant effect (Figure 4C; Supplementary Figure 7A).

Next, we inhibited neural activities in the OSNs by expressing Kir2.1, because the avoidance of 2MT was altered in *Orco* mutant flies, particularly at low concentrations (Figures 2A, D, E). Electrical silencing of the OSNs by *Orco*-*GAL4* abolished the avoidance of 100 μM 2MT and partially impaired the avoidance of 1 mM 2MT (Figures 4D, E; Supplementary Figure 7B), consistent with the results obtained from *Orco* mutant flies (Figures 2A, D). These results indicate that adult flies detect 2MT through TrpA1 expressed in bitter taste neurons and nociceptive neurons, in conjunction with ORs expressed in OSNs.

### TrpA1-C and TrpA1-D isoforms play key role in aversive responses to 2MT

**FIGURE 5:**
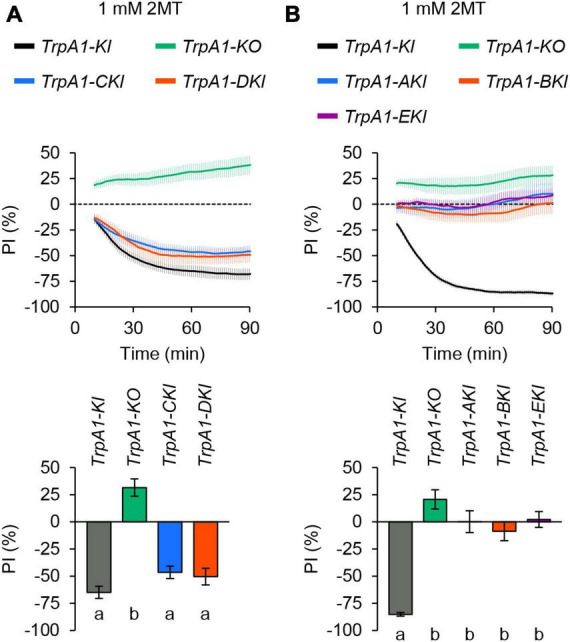
TrpA1-C and TrpA1-D isoforms were involved in 2MT avoidance. The temporal changes of PI to 1 mM 2MT (top) and the PI at 60 min (bottom). A dotted line in the top panel represents the level indicating no preference or avoidance. The data are presented as moving average ± SEM. **(A)** The PI to 1 mM 2MT in *TrpA1^1^*/*TrpA1-T2A-GAL4* (*TrpA1-KI*; black), *TrpA1^1^*/*TrpA1-KO* (*TrpA1-KO*; green), *TrpA1^1^/TrpA1-CKI-T2A-GAL4* (*TrpA1-CKI*; blue), and *TrpA1^1^*/*TrpA1-DKI-T2A-GAL4* (*TrpA1-DKI*; red) flies. The same letters indicate no significant difference based on one-way ANOVA with Tukey’s multiple comparison (*N* = 12–16). **(B)** The PI to 1 mM 2MT in *TrpA1-KI* (black), *TrpA1-KO* (green), *TrpA1^1^*/*TrpA1-AKI-T2A-GAL4* (*TrpA1-AKI*; blue), *TrpA1^1^*/*TrpA1-BKI-T2A-GAL4* (*TrpA1-BKI*; red), and *TrpA1^1^*/*TrpA1-EKI-T2A-GAL4* (*TrpA1-EKI*; purple) flies. The same letters indicate no significant difference based on Kruskal-Wallis test with Steel-Dwass multiple comparison (*N* = 12–21).

*Drosophila melanogaster* TrpA1 has five different splicing variants: TrpA1-A, TrpA1-B, TrpA1-C, TrpA1-D, and TrpA1-E (Zhong et al., 2012; Gu et al., 2019). While all isoforms except TrpA1-E respond to the wasabi ingredient AITC when expressed in tissue culture cells, they have different contributions to thermal and chemical sensation in larvae (Zhong et al., 2012; Gu et al., 2019) and adult flies (Kang et al., 2011; Du et al., 2015; Leung et al., 2020). To determine which TrpA1 isoforms are essential for 2MT avoidance, we used TrpA1 isoform-specific knock-in (KI) alleles (Gu et al., 2019), which allow the expression of a single TrpA1 isoform by mutating exons specific to other isoforms. Heterozygotes of the KI or KO alleles were generated by crossing with the *TrpA1^1^* null mutant to ensure the viability of some unhealthy homozygous KI alleles. Heterozygotes of *TrpA1-CKI* and *TrpA1-DKI* displayed strong avoidance behavior to 2MT, similar to *TrpA1-KI* heterozygote that expresses all isoforms (Figure 5A; Supplementary Figure 8A). Conversely, *TrpA1-AKI*, *TrpA1-BKI*, and *TrpA1-EKI* heterozygous flies did not exhibit avoidance of 2MT at the same concentration, resembling the response of *TrpA1-KO/TrpA1^1^* transheterozygotes (Figure 5B; Supplementary Figure 8B). These results indicate that the expression of either the TrpA1-C or TrpA1-D isoform is sufficient to respond to 2MT in adult flies.

### TrpA1-C and TrpA1-D isoforms are expressed in the sensory neurons in the legs

To determine the expression pattern of TrpA1-C and TrpA1-D isoforms in chemosensory and nociceptive neurons, we examined the expression of membrane-tethered green fluorescent protein (mCD8::GFP) induced in TrpA1 isoform-specific KI lines. These KI lines carried a *T2A*-*GAL4* sequence just upstream of the stop codon (Gu et al., 2019), allowing GAL4 expression in isoform-specific cells. Since the expression patterns in the proboscis and maxillary palps have been previously described (Gu et al., 2019), we investigated the expression patterns of TrpA1-C and TrpA1-D in sensory neurons in the legs and labellum.

**FIGURE 6:**
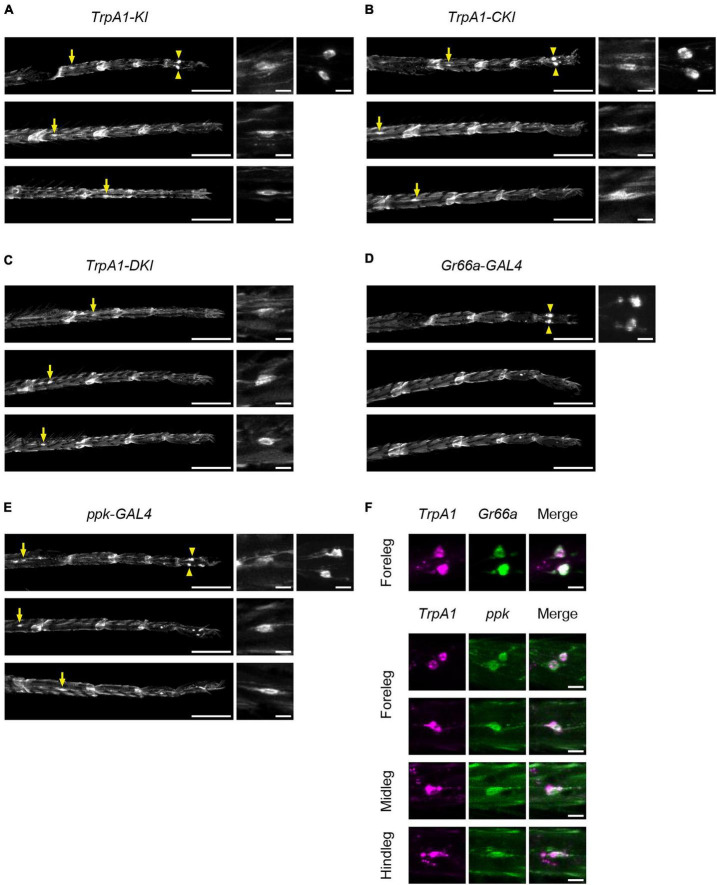
TrpA1-C and TrpA1-D isoforms were expressed in the sensory neurons in the legs. **(A–E)** The GFP expression in the forelegs (top), the midlegs (middle), and the hindlegs (bottom). Arrows indicate cell bodies of *ppk*-neurons located in the 1st–3rd segments of tarsus. Arrowheads indicate cell bodies of *ppk*-neurons and *Gr66a*-neurons located in the 5th segment of protarsus in the forelegs. Magnified images of cell bodies are shown on the right. **(A)** *TrpA1-T2A-GAL4*/*UAS*-*mCD8::GFP*. **(B)** *TrpA1-CKI-T2A-GAL4*/*UAS*-*mCD8::GFP*. **(C)** *TrpA1-DKI-T2A-GAL4*/*UAS*-*mCD8::GFP*. **(D)** *Gr66a-GAL4*/*UAS*-*mCD8::GFP*. **(E)** *ppk-GAL4*/*UAS*-*mCD8::GFP*. Scale bars represent 100 μm (left) or 10 μm (right). **(F)** The mCherry (left), GFP (middle), and merged (right) images in the legs. Magnified images of *Gr66a*-neurons in the forelegs in *lexAop2*-*mCherry*/*Gr66a-GAL4*; *TrpA1-T2A-LexA*/*UAS*-*mCD8::GFP* (top). Magnified images of *ppk*-neurons in the forelegs, midlegs, and hindlegs in *lexAop2*-*mCherry*/*UAS*-*mCD8::GFP*; *TrpA1-T2A-LexA*/*ppk-GAL4* (bottom). Scale bars represent 10 μm. The expression patterns in all samples were confirmed in three individuals.

First, we observed *TrpA1-T2A*-*GAL4* (*TrpA1-KI*), which expresses all isoforms, to determine the overall TrpA1 expression pattern. In the forelegs, two large cell bodies located in the fifth segments of protarsus were clearly labeled, similar to the pattern of *Gr66a*- and *ppk*-positive neurons (Figures 6A, D, E; Supplementary Figure 9A). This expression was also observed in *TrpA1-CKI-T2A*-*GAL4* and *TrpA1-BKI-T2A*-*GAL4* flies but not in *TrpA1-DKI-T2A*-*GAL4* and *TrpA1-AKI-T2A*-*GAL4* flies (Figures 6B, C; Supplementary Figures 9B, C). *TrpA1-KI* flies also exhibited weak GFP signals in neurons with small cell bodies located in the proximal segments of the tarsus in the forelegs, midlegs, and hindlegs. The position and shape resembled those of *ppk-GAL4*-expressing nociceptive neurons (Figures 6A, E; Supplementary Figure 9A). This *ppk*-like expression pattern was observed in both *TrpA1-CKI-T2A*-*GAL4* and *TrpA1-DKI-T2A*-*GAL4* flies (Figures 6B, C), but not in *TrpA1-AKI-T2A*-*GAL4* or *TrpA1-BKI-T2A*-*GAL4* flies (Supplementary Figures 9B, C). To confirm TrpA1 expression in gustatory and nociceptive neurons, we crossed *TrpA1-T2A-LexA*, which carries a red fluorescent protein (mCherry) expressed in all isoform-expressing cells, with *Gr66a-GAL4* or *ppk-GAL4*, expressing mCD8::GFP in these neurons (Figure 6F). As anticipated from individual labeling (Figures 6A, D, E), TrpA1 co-localized with Gr66a and Ppk in all legs (Figure 6F). We also examined the expression patterns of *Gr66a*- and *ppk*-*GAL4* in the labellum alongside *TrpA1-T2A-LexA*. TrpA1 showed widespread expression in gustatory neurons and co-localization within a subset of Gr66a neurons (Supplementary Figure 10A). However, co-expression of Ppk and TrpA1 was not observed in the labellum (Supplementary Figure 10B). Based on these results and the behavioral outputs in Figure 4, we suggest that bitter taste neurons expressing TrpA1-C and nociceptive neurons expressing TrpA1-C and TrpA1-D contribute to aversive responses to 2MT.

### TrpA1-C and TrpA1-D isoforms are activated by 2MT through the evolutionarily conserved cysteine residues

**FIGURE 7:**
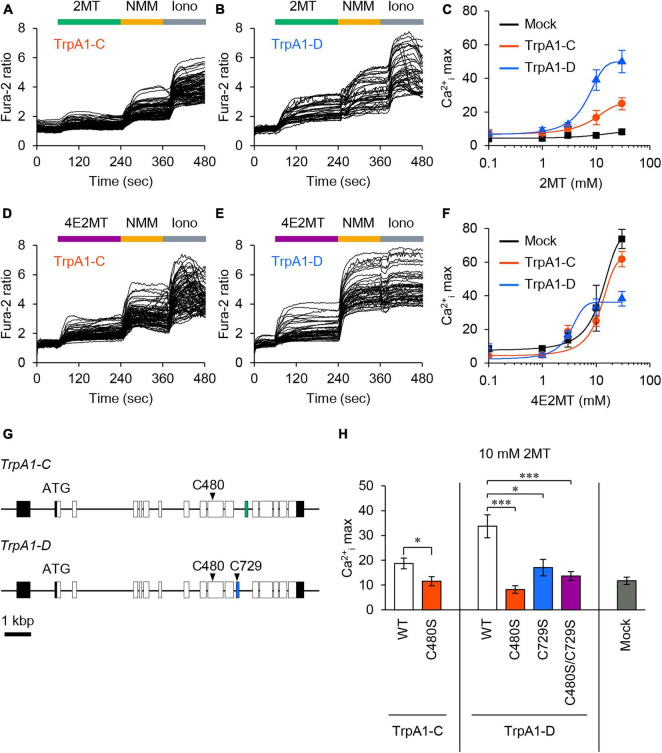
2MT activated TrpA1-C and TrpA1-D isoforms through cysteine modification. **(A,B,D,E)** Representative Fura-2 responses to 2MT **(A,B)** or 4E2MT **(D,E)** are shown in cells expressing TrpA1-C **(A,D)** or TrpA1-D **(B,E)**. S2R+ cells were stimulated with 10 mM 2MT (green bar) or 10 mM 4E2MT (purple bar). To assess the channel function, 100 μM NMM (yellow bar) was applied. Cell viability was confirmed by the application of 2.5 μM ionomycin (Iono; gray bar). **(C,F)** Fura-2 dose-response curves of TrpA1-C (red), TrpA1-D (blue), and mock control (black) to 2MT **(C)** or 4E2MT **(F)**. The data are presented as mean ± SEM. **(G)** The genomic structure of *TrpA1-C* and *TrpA1-D* isoforms. Coding and non-coding exons are represented by white and black boxes, respectively, and alternatively spliced exons by green and blue boxes. **(H)** Quantification of the maximal Ca^2+^_i_ increase (Ca^2+^_i_ max) in response to 10 mM 2MT in cells expressing TrpA1-C (left, white), TrpA1-C C480S (left, red), TrpA1-D (middle, white), TrpA1-D C480S (middle, red), TrpA1-D C729S (middle, blue), TrpA1-D C480S/C729S (middle, purple), and mock control (right, black). The data are presented as mean ± SEM (*N* = 6–13). *P < 0.05; ***P < 0.001 by Student’s *t*-test or Kruskal-Wallis test with Steel multiple comparison.

To investigate the responsiveness of *Drosophila* TrpA1-C and TrpA1-D isoforms to 2MT, we conducted *in vitro* Ca^2+^ imaging in S2R+ cells expressing either TrpA1-C or TrpA1-D. The application of 2MT resulted in dose-dependent increases in intracellular Ca^2+^ ([Ca^2+^]_i_) levels, specifically in cells expressing TrpA1-C or TrpA1-D, whereas control cells did not exhibit such responses (Figures 7A–C). Notably, TrpA1-D showed a more potent increase in [Ca^2+^]_i_ than TrpA1-C. We also observed [Ca^2+^]_i_ increases in TrpA1-C and TrpA1-D-expressing cells upon exposure to 4E2MT, although non-specific effects of the compound became evident at higher concentrations, and [Ca^2+^]_i_ increases were indistinguishable between TrpA1-expressing cells and control cells (Figures 7D–F).

Previous studies have demonstrated that 2MT activated mouse TRPA1 by covalently modifying six cysteine residues (Wang Y. et al., 2018). In *Drosophila* TrpA1, two cysteine residues are conserved: C480 and C729 in TrpA1-D (corresponding to C415 and C666 in mice, respectively), and C480 in TrpA1-C (corresponding to C415 in mice) (Figure 7G; Supplementary Figure 11). To assess the role of these conserved cysteine residues in *Drosophila* TrpA1, we substituted each residue with serine in TrpA1-C (C480S) and TrpA1-D (C480S, C729S, and C480S/C729S). Mutating single or multiple cysteine residues in these isoforms resulted in a significant reduction in [Ca^2+^]_i_ responses to 2MT, almost reaching the background level (Figure 7H). Importantly, we confirmed that the double mutant in TrpA1-D retained responsiveness to citronellal, which activates the channel through non-covalent mechanisms (Du et al., 2015; Boonen et al., 2021), indicating that the mutated channels remained functional (Supplementary Figure 12). These results strongly suggest that 2MT activates *Drosophila* TrpA1 by specifically interacting with these conserved cysteine residues.

## Discussion

In this study, we characterized the effect of the thiazoline-related compound 2MT on behavioral responses in both male and female *Drosophila melanogaster* and investigated the underlying mechanisms. The aversive responses to high concentrations (300 μM–3 mM) of 2MT were primarily mediated by gustatory and nociceptive pathways through TrpA1, whereas the avoidance behaviors in response to low concentrations (<300 μM) of 2MT were mediated by olfactory pathways involving ORs. Unlike bitter substances, which typically exert their effects through the gustatory pathway in proboscis, 2MT demonstrates its repulsive effects even with minimal feeding involvement (Figure 1). This highlights its potential as a promising lead chemical for developing insect repellents. Furthermore, even in *TrpA1* mutants, higher concentrations of 2MT (> 3 mM) elicited clear aversive responses (Figure 2), suggesting the existence of additional receptors and detection mechanisms for 2MT.

TrpA1 is expressed in taste neurons, nociceptive neurons, and OSNs in *Drosophila* (Kang et al., 2010, 2011; Kim et al., 2010; Kwon et al., 2010; Khuong et al., 2019; Leung et al., 2020). Knocking down *TrpA1* in taste or nociceptive neurons reduced the avoidance response to 2MT, but not in OSNs (Figure 4), indicating the functional role of TrpA1 in contact chemosensory pathways. Conversely, mutation of *Orco* and neural silencing of OSNs using Kir2.1 led to reduced 2MT avoidance, indicating the contribution of the olfactory pathway to 2MT avoidance (Figures 2, 4). Notably, a previous study in mice demonstrated similar characteristics of the effect of 2MT: TRPA1 expressed in the trigeminal and vagus nerves mediates 2MT responses, whereas TRPA1 expressed in OSNs does not (Matsuo et al., 2021a). Nevertheless, OSNs are also necessary for the 2MT response (Matsuo et al., 2021a). Like 2MT, other TRP channel activators drive multiple sensory pathways in insects. Flies detect naturally occurring insect repellent citronellal through ORs, TrpA1 expressed in OSNs and taste neurons, and Trpγ in OSNs (Kwon et al., 2010; Du et al., 2015; Tian et al., 2022). The repellent effect of camphor is mediated by Trpl expressed in taste neurons and ORs (Zhang et al., 2013; Wang Q. et al., 2021), and the repulsion caused by menthol involves TrpA1 and ORs (Boonen et al., 2021; Wang Q. et al., 2021). Collectively, we propose that multiple sensory processes contribute to the detection of 2MT and subsequent aversive responses, and such mechanisms may present key features of TRP channel activators as insect repellents.

Interestingly, *Orco* mutant flies displayed attractive responses, rather than aversive responses, to low concentrations (30, 100, and 300 μM) of 2MT (Figure 2; Supplementary Figure 4). These paradoxical effects of 2MT in mutant flies suggest that 2MT is detected not only as an aversive cue but also as an attractive cue by unidentified chemosensory receptors in flies. 2-Methylthiazolidine, structurally similar to 2MT, is one of the components of male sex pheromones in cockroaches (*Nauphoeta cinerea*) and robustly attracts females at very low concentrations (Sirugue et al., 1992). 2MT also has an attractive effect on cockroach females, although the trace amount found in male’s glands is insufficient to exert attractiveness. These findings might support the possible existence of unknown chemoreceptors in flies involved in attractive behavior toward 2MT at low concentrations. In the labellum, TrpA1 is present in both bitter neurons and other taste neurons (Supplementary Figure 10). Neurons exclusively expressing TrpA1, distinct from Gr66a, could potentially contribute to the mild attraction to 2MT in *Drosophila*.

We found that other thiazoline-related chemicals, 4E2MT and TMO, evoked avoidance responses (Figure 3). These chemicals activate mouse TRPA1 and induce antihypoxic effects similar to 2MT in mice (Matsuo et al., 2021a). However, unlike 2MT, TMO-induced avoidance was not reduced in *TrpA1* mutant flies (Figure 3), suggesting that flies rely on TrpA1-independent pathways to detect TMO. In contrast, 4E2MT-induced avoidance was not altered in *Orco* mutant flies and was partially reduced in *TrpA1* mutant flies (Figure 3). Additionally, 4E2MT elicited non-specific Ca^2+^ responses in mock-transfected *Drosophila* cells that did not express TrpA1 (Figure 7). These findings imply that 4E2MT acts on unknown receptors or non-specifically on *Drosophila* cells.

There are five isoforms of TrpA1 in *Drosophila melanogaster*. All isoforms are expressed in pharyngeal taste neurons (Gu et al., 2019). TrpA1-C and TrpA1-D are reported to be expressed in taste neurons in the labellum, whereas TrpA1-A, TrpA1-B, and TrpA1-E isoforms are not or less expressed. We observed TrpA1-C and TrpA1-D expression in chemosensory and/or nociceptive neurons in the legs (Figure 6). We found that 2MT avoidance was only observed in *TrpA1-CKI* and *TrpA1-DKI* flies, which express the TrpA1-C and TrpA1-D isoforms, respectively, but not in *TrpA1-AKI*, *TrpA1-BKI*, and *TrpA1-EKI* flies (Figure 5). The different expression patterns may reflect the differential functions of the isoforms in 2MT detection. It is noteworthy that TrpA1-B isoform was weakly expressed in *Gr66a*-bitter taste neurons, similar to the TrpA1-C isoform (Supplementary Figure 9). However, unlike TrpA1-C, TrpA1-B did not contribute to 2MT avoidance (Figure 5). This similar expression pattern might be due to a shared alternative exon in these isoforms (Zhong et al., 2012; Gu et al., 2019). Consistent with this notion, TrpA1-C and TrpA1-D isoforms were expressed in *ppk*-nociceptive neurons in the forelegs, midlegs, and hindlegs (Figure 6), and these isoforms share the first exon (Zhong et al., 2012; Gu et al., 2019). The expression pattern of each isoform might depend on specific exons/introns.

Previous studies have demonstrated differences in chemical sensitivity among *Drosophila* TrpA1 isoforms. The sensitivities of TrpA1-C and D to citronellal are higher than that of TrpA1-A, while TrpA1-A is more responsive to menthol than TrpA1-C and D (Du et al., 2015; Boonen et al., 2021). TrpA1-B is the least sensitive to both citronellal and menthol (Boonen et al., 2021). Aristolochic acid, a phytochemical, activates TrpA1-D but not TrpA1-C (Leung et al., 2020). Nepetalactone, a natural repellent in catnip, activates TrpA1-C but not TrpA1-A (Melo et al., 2021). Furthermore, TRPA1 chemical sensitivity also differs among isoforms in mosquitoes, such as *Anopheles gambiae*, *Anopheles stephensi*, *Aedes aegypti*, and *Culex pipiens pallens* (Du et al., 2015; Li et al., 2019; Melo et al., 2021). Taken together, the physiological role of each isoform likely depends on the variations in chemical sensitivity and expression patterns.

Mammalian TRPA1 is activated by electrophiles through covalent modification of specific cysteine residues, and *Drosophila* TrpA1 uses evolutionarily conserved cysteine residues to respond to electrophiles (Hinman et al., 2006; Macpherson et al., 2007; Kang et al., 2010). A previous study identified six cysteine residues involved in the activation of mouse TRPA1 by 2MT (Wang Y. et al., 2018). Among them, two cysteine residues (C480 and C729) are conserved in fly TrpA1: C480 and C729 in TrpA1-D and C480 in TrpA1-C (Figure 7; Supplementary Figure 11). The Ca^2+^ responses of TrpA1-D were more potent than TrpA1-C, and a single mutation of C729 resulted in a reduction of 2MT-induced activation (Figure 7). This mutant retains another cysteine (C480) and the Ca^2+^ response was comparable to that in TrpA1-C isoform that carries C480. C480 substitution in TrpA1-C and the double cysteine mutation in TrpA1-D resulted in a reduction in Ca^2+^ responses to the background level, indicating that these cysteine residues are essential for 2MT-dependent channel activation. C480 and C729 exhibit a high degree of conservation in TRPA1 across a wide range of insect species, including agricultural pests and disease vectors such as moths (*Bombyx mori*, *Bombyx mandarina*, *Manduca sexta*, *Tuta absoluta*, *Galleria mellonella*, and *Helicoverpa armigera*), aphid (*Acyrthosiphon pisum*), mosquitoes (*Anopheles gambiae*, *Aedes aegypti*, and *Culex pipiens*) (Kang et al., 2010; Kwon et al., 2010; Wei et al., 2015; Wang X. D. et al., 2021), as well as spotted wing drosophila (*Drosophila suzukii*), cockroach (*Blattella germanica*), planthoppers (*Sogatella furcifera* and *Nilaparvata lugens*), striped riceborer (*Chilo suppressalis*), whitefly (*Bemisia tabaci*), and lygus bug (*Lygus hesperus*) (Supplementary Figure 13). Based on this, we anticipate that the aversive effect of 2MT could be preserved among these species. Further research should investigate the impact of 2MT on these insects at both the behavioral and molecular levels.

Our results suggest that insect TRP channels could serve as promising targets for developing repellents and insecticides. A recent study identified commercial insecticides, such as pymetrozine and pyrifluquinazon, as activators of insect TRPV channels–Inactive and Nanchung–which are expressed in stretch receptor cells (Nesterov et al., 2015). These synthetic chemicals induce insecticidal effects by disrupting normal locomotive and feeding behaviors in insect pests. Additionally, we propose that 2MT holds significant potential as an insect repellent for several reasons. Firstly, it functions through both olfaction and contact chemosensation. Secondly, mere contact with 2MT effectively triggers aversion without the need for feeding. Notably, a previous report did not detect activation of human TrpA1 by 2MT despite the conservation of four critical cysteines (Wang Y. et al., 2018). It is also worth mentioning that 2MT has been commercially used as a food additive, although the concentration range is generally 0.1–10 ppm (Fernandez et al., 2002). Considering these findings along with our results, it becomes evident that 2MT and related chemicals have the potential to be valuable resources in development of novel insect repellents and insecticides, with insect TRP channels as promising targets.

## Materials and methods

### Fly stocks

Flies were reared on glucose-yeast-cornmeal media: 2,500 ml of reverse osmosis water, 180 g of cornmeal (Oriental Yeast, Tokyo), 100 g of dry brewer’s yeast Ebios (#128-297405, Mitsubishi Tanabe Pharma, Osaka), 19 g of agar (#RSU-AL01, RIKAKEN, Tokyo), 250 g of glucose (#TDH, San-ei Sucrochemical, Aichi), 24 ml of methyl 4-hydroxybenzoate (10% in 70% ethanol; #H5501, Sigma-Aldrich, MA), and 8 ml of propionic acid (#81910, Sigma-Aldrich, MA). Flies were raised in vials or bottles at 25°C under 12-h light/12-h dark cycle. *w*^1118^ was outcrossed for ten generations to the wild-type Canton-S (CS) genetic background and used as a control. The fly lines used in this study are as follows: *TrpA1^1^* [Bloomington stock center (BL, IN) #26504], *Orco*^2^ (BL #23130), *wtrw*^2^ (BL #59038), *Trp^MB03672^* (BL #23636), *Trpl^MB10553^* (BL #29134), *Trpγ^G4^* (BL # 64313), *Orco*-*GAL4* (BL #26818), *UAS*-*dicer2* (BL #24650), *UAS*-*TrpA1* RNAi (BL #36780), *UAS*-*Kir2.1::GFP* (KYOTO Drosophila Stock Center, Kyoto #108846), *UAS*-*mCD8::GFP* (BL #5137 and #32195). The following stocks were provided by the indicated investigators: *TrpA1-T2A-GAL4*, *TrpA1-KO*, *TrpA1-AKI-T2A-GAL4*, *TrpA1-BKI-T2A-GAL4*, *TrpA1-CKI-T2A-GAL4*, *TrpA1-DKI-T2A-GAL4*, *TrpA1-EKI-T2A-GAL4*, and *lexAop2*-*mCherry*; *TrpA1-T2A-LexA* (Dr. Y. Xiang) (Gu et al., 2019), *Gr66a*-*GAL4* (Dr. H. Amlein), *ppk*-*GAL4* B-3207d (Dr. D. N. Cox), and *pain*^4^ (Dr. C. Montell) (Liu et al., 2023). All lines except the *UAS*-*dicer2*, *UAS*-*TrpA1* RNAi, *UAS*-*mCD8::GFP*, *TrpA1-T2A-GAL4*, *TrpA1-KO*, *TrpA1-KI-T2A-GAL4*, and *lexAop2*-*mCherry*; *TrpA1-T2A-LexA* lines were outcrossed to *w*^1118^ flies on the CS genetic background for five generations.

### Chemicals

The following chemicals were purchased from Tokyo Chemical Industry (Tokyo): quinine hydrochloride dihydrate (#Q0030), 2-methylthiazoline (2MT; #M0285), 2-methyl-4-ethylthiazoline (4E2MT; #M0689), thiomorpholine (TMO; #T1007), and 2-methyl-2-oxazoline (2MO; #M0857). *N*-methylmaleimide (NMM; #389412) and citronellal (#373753) were purchased from Sigma-Aldrich (MA). Quinine was dissolved in water and kept at −20°C. 2MT, TMO, and 2MO were dissolved in water just before use for behavioral assays. 4E2MT was dissolved in dimethyl sulfoxide (DMSO) just before use for behavioral assays. 2MT and 4E2MT were dissolved in a bath solution and sonicated for 1 min in an ultrasonic cleaner (#MCS-2P, AS ONE Corporation, Osaka) just before use (see below for the composition of the bath solution). NMM was dissolved in DMSO and kept at −20°C.

### Two-choice positional preference assay

The assay was conducted following a similar protocol as previously described (Sanchez-Alcaniz et al., 2017). Male flies (3–8 days old) were used for all behavioral assays. A total of fifty adult males were collected and kept in vials containing standard food until the onset of starvation. To induce starvation, flies were deprived of food and maintained in vials containing 1 ml of 1% agarose for 24 h prior to the assay. Non-starved flies were transferred to vials containing food 24 h before the experiment.

For the assay plates, we used 100-mm diameter four-section petri dishes (#25384-308, VWR, PA) along with custom-made quadrant acrylic blocks. Each quadrant of the petri dish was fitted with the acrylic block, and a small amount of 1% agarose was added to fill the gap. After the agarose has solidified, the surface of the acrylic block in each quadrant was covered with 0.5% agarose containing either a test chemical or a solvent (1.2 ml per quadrant). Unless otherwise stated, all quadrants contained 2 mM sucrose. The test chemicals were alternately present or absent in each quadrant. If the chemical was dissolved in DMSO, the final concentration of DMSO was 0.1%. Flies in vials were gently anesthetized with ice for up to 70 s and immediately transferred to an assay plate. The assay plates were covered with lids and placed upside down on an acrylic stage, allowing the flies come into contact with the agarose through negative geotaxis. The acrylic stage was elevated 5 cm from an LED light tracer (6800 lux; SV531A, Sinkosha, Tokyo), with the LED light covered with a red film (#105, LEE Filters, Hampshire). The assay chamber was covered with a light shielding curtain. Still images of the assay plates were captured from the top every 1 min using a digital camera (FDR-AX60, Sony, Tokyo) for a duration of up to 120 min. All behavioral experiments were initiated in the morning [zeitgeber time (ZT) 1.5–2]. See Supplementary Figure 1 for a schematic of the setup.

The number of flies In each quadrant were quantified using a custom macro in ImageJ (Sanchez-Alcaniz et al., 2017) with some modifications. The preference index (PI) was calculated using the following formula: PI (%) = (N_chemical_ – N_control_)/(N_chemical_ + N_control_) × 100, where N_chemical_ and N_control_ represent the number of flies in the chemical-containing quadrants and control quadrants, respectively. PIs were calculated over time using 21-min moving averages.

### Oviposition assay

We used female flies aged 4–8 days old. A total of 30–40 adult females and ten adult males were collected and kept in vials containing standard food. Four days prior to the assay, the flies were transferred to food vials containing yeast paste. The preparation of assay plates followed a similar procedure as described above for the two-choice positional preference assay. To enhance egg production, we coated the surface of the acrylic blocks in quadrants with soft agarose (0.25%) containing 10 mM sucrose. Five to eight female flies were gently transferred onto each assay plate using an aspirator. The assay plates were then placed in a dark box with water-soaked paper. Females were allowed to lay eggs from the morning (ZT 2–2.5) to the evening (ZT 8–8.5) at a temperature of 25°C.

The number of eggs in each quadrant was manually counted. Oviposition index (OI) was calculated using the following formula: OI (%) = (E_chemical_ – E_control_)/(E_chemical_ + E_control_) × 100, where E_chemical_ and E_control_ represent the number of eggs in the quadrants with the chemical and control quadrants, respectively. Trials in which the total number of eggs laid was fewer than twenty were excluded from the analyses.

### Measurement of locomotor activity

Assay plates were prepared using a 96-well microplate (#1-1601-02, VIOLAMO, Osaka). We applied 180 μl of 1% agarose with or without test chemicals to each well. Once the agarose has solidified, we sealed the plate with plate seal (#547-SBS-PET, WATSON, Tokyo) and cut a cross slit over each well. Male flies (3–8 days old) were individually transferred into each well using an aspirator. The flies were allowed to move freely within the wells and were recorded using a digital camera (FDR-AX60, Sony, Tokyo) in the assay chamber, similar to the two-choice positional preference assay as described above. The recordings were captured at a frame rate of 30 frames per second for 10 min at 0, 30, and 60 min after placing the assay plate in the chamber. All behavioral experiments were conducted in the afternoon (ZT 5.5–6.5). We measured the total distance moved (mm) as an index of locomotor activity (Sakai et al., 2009), which was calculated using the video tracking software Move-tr/2D (version 8.4, Library, Tokyo).

### Confocal microscopy

For observation of the expression pattern of TrpA1 isoforms, *UAS*-*mCD8::GFP* was crossed with *TrpA1-T2A-GAL4*, *TrpA1-AKI-T2A-GAL4*, *TrpA1-BKI-T2A-GAL4*, *TrpA1-CKI-T2A-GAL4*, *TrpA1-DKI-T2A-GAL4*, *Gr66a*-*GAL4*, and *ppk*-*GAL4* B-3207d. For observation of the co-expression of TrpA1 with *Gr66a*- and *ppk*-*GAL4*, *lexAop2*-*mCherry*; *TrpA1-T2A-LexA* was crossed with *Gr66a*-*GAL4*; *UAS*-*mCD8::GFP* and *UAS*-*mCD8::GFP*; *ppk*-*GAL4*. We dissected forelegs, midlegs, hindlegs, and labellum from male flies (4–10 days old) in phosphate-buffered saline (PBS), and mounted the samples in 50% glycerol/PBS. GFP and mCherry fluorescence and brightfield images were acquired using a confocal laser scanning microscope (FV1200, Olympus, Tokyo) and a 30 × /1.05 UPLSAPO30XSIR dry objective lens. Z-sections were taken at a 1-μm interval. Images were analyzed using FLUOVIEW (version 4.2c, Olympus, Tokyo) and Fiji software (Schindelin et al., 2012).

### Cloning

For the cloning of *TrpA1-C* and *TrpA1-D* isoforms, we conducted RT-PCR using total RNA extracted from *w*^1118^ third instar larvae following the manufacturer’s protocol. We prepared 13 μl reaction premix containing 50 μM Oligo(dT)_20_, 10 mM dNTP mix, 5 mg of total RNA in DEPC-treated water in a 0.2-ml PCR tube. The premix was incubated at 65°C for 5 min, and chilled on ice for over 1 min. Subsequently, the incubated reaction premix was combined with 7 μl of mix containing 4 μl of 5X SuperScript IV RT buffer, 1 μl of 100 mM DTT, 1 μl of SuperScript™ IV Reverse Transcriptase (#18090010, Invitrogen, MA), and 1 μl of RNaseOUT™ Recombinant Ribonuclease Inhibitor (#10777019, Invitrogen, MA). The reaction mix was incubated at 55°C for 10 min followed by 80°C for 10 min. The RT product was stored in −20°C until further use.

To amplify *TrpA1-C* and *TrpA1-D* cDNAs, we performed PCR using a common forward primer containing the KOZAC sequence upstream of the start codon and a common reverse primer (Supplementary Table 1). Phusion High-Fidelity DNA Polymerase (#M0530S, Invitrogen, MA) was mixed with 2 μl (∼500 ng) of undiluted RT template in 50 μl reaction. The PCR protocol consisted of an initial denaturation step at 98°C for 30 s, followed by 35 cycles of denaturation at 98°C for 10 s, annealing at 66°C for 30 s, and extension at 72°C for 2 min and 40 s. The final extension was performed at 72°C for 2 min. The PCR product and pAc5.1-V5-His A vector (#V411020, Invitrogen, MA) were digested with *Not*I-HF [#R3189S, New England Biolabs (NEB), MA] and *Xba*I (#R0145S, NEB, MA). The digested PCR product and vector were ligated using Ligation High Ver.2 (#LGK-201, TOYOBO, Osaka) at 16°C for 30 min and then transformed into *DH5α* competent cells. *E. coli* culture were plated on LB agar (#20067-85, nacalai tesque, Kyoto) containing 100 μg/mL Ampicillin (#016-23301, Wako, Osaka) in a 10-cm petri dish (#SH90-15, IWAKI, Shizuoka) and incubated overnight at 37°C. The colonies were inoculated into Plusgrow II (#08202-75, nacalai tesque, Kyoto) containing 100 μg/mL Ampicillin and cultured overnight in a 37°C shaker. The plasmid was extracted using NucleoSpin Plasmid EasyPure (#U0727C, Takara, Shiga). The presence of *dTrpA1-C* or *dTrpA1-D* in the constructs was confirmed by digestion with *Ssp*I-HF (#R3132S, NEB, MA), resulted in the following fragments: *TrpA1-C/*pAc5.1-V5-His A: 4063, 2751, 992, 637, 321, and 284 bp; *TrpA1-D/*pAc5.1-V5-His A: 6817, 992, 637, 321, and 284 bp. The entire sequences were confirmed (Supplementary Figure 14). The plasmids were amplified using NucleoBond Xtra Midi (740410.50, Macherey-Nagel, Düren) and stored at −20°C.

### Site-directed mutagenesis

We performed PCR to substitute cysteines at position 480 and/or 729 with serine in wild-type *TrpA1-C*/pAc5.1-V5-His A or *TrpA1-D*/pAc5.1-V5-His A. The cysteine codon in the region of interest was replaced with serine using a pair of overlapping oligonucleotides containing the designated mutation. The synthetic oligonucleotide primers carrying the mutations are listed in Supplementary Table 2.

A two-step PCR reaction was performed using Phusion High-Fidelity DNA Polymerase (#M0530S, NEB, MA). The reaction premix contained 200 μM dNTPs, 10 μM each of forward and reverse oligonucleotides, 200 pg of template DNA, 0.2 units of Phusion High-Fidelity DNA Polymerase dissolved in 5X Phusion buffer and MilliQ water. The PCR protocol consisted of an initial denaturation step at 98°C for 30 s, followed by 25 cycles of denaturation at 98°C for 10 s and annealing/extension at 72°C for 3 min. The final extension was performed at 72°C for 5 min. The amplified plasmid was subjected to demethylation using *Dpn*I for 1 h at 37°C, and transformed into *DH5α E. coli* strain for overnight culture. The resulting colonies were purified using NucleoBond Xtra Midi (740410.50, Macherey-Nagel, Düren) for subsequent experiments.

### Ca^2+^ imaging

Schneider 2 R+ (S2R+) cells were grown in 60-mm dishes (#353002, Falcon, NY) at 25°C in 4 ml of Schneider’s medium (#21720-024, Gibco, MA) containing 10% inactivated fetal bovine serum (#10437-028, Gibco, MA) and 50 mg/mL penicillin/50 units/mL streptomycin (#15140-122, Gibco, MA). For a transient transfection, a mix was prepared with 1 μg of *TrpA1-C*, *TrpA1-D*, or the mutants of each isoform in a pAc5.1-V5-His expression vector and 0.1 μg of *DsRed*/pAc5.1-V5-His at the 10:1 ratio using X-tremeGENE9 Transfection Reagent (#06365787001, Roche, Basel) dissolved in 1X OPTI-MEM medium (#31985-070, Thermo Fisher, MA), following the manufacturer’s protocol. The transfection mix was incubated for 15 min at room temperature. S2R+ cells were reseeded onto 12-mm cover glasses (Matsunami, Osaka) in a 35-mm dishes (#1000-035, IWAKI, Shizuoka) in 2 ml of culture media and the transfection mix was added to the media. The cells were incubated for 48 h in a 25°C incubator. To load the cells with Fura-2 AM, we added 1 mL of the following mixture to the culture media and incubated the cells in a 25°C incubator for 1–3 h: 5 μM Fura-2 AM (#F-1201, Life Technologies, MA), 1 mM probenecid (#162-26112, Wako, Osaka), and 0.02% pluronic F-127 detergent (#P2443, Sigma-Aldrich, MA). The cover glasses were mounted in a chamber (#RC-26G; Warner Instruments, MA) filled with a bath solution containing 130 mM NaCl, 5 mM KCl, 2 mM MgCl_2_, 2 mM CaCl_2_, 30 mM sucrose, and 10 mM N-Tris(hydroxymethyl)methyl-2-aminoethanesulfonic acid (TES), after adjusting the pH to 7.2 with NaOH. 500 μM probenecid was dissolved in the bath solutions to retain Fura-2 in the cells. The chamber was connected to a gravity flow system to deliver various stimuli. A xenon lamp was used as an illumination source. To obtain fluorescent intensities of Ca^2+^-bound and Ca^2+^-free Fura-2, we excited the cells at 340 and 380 nm, respectively, and emission was monitored at 510 nm with a CCD camera, CoolSNAP DYNO (Photometrics, AZ) used with a fluorescent microscope (#TE-300, Nikon, Tokyo). Data was measured using the NIS element AR (Nikon, Tokyo).

We analyzed DsRed-positive cells and obtained a trace for each cell using the NIS element AR. The change in Ca^2+^_i_ was calculated as follows: Ca^2+^_i_ response = (F_res_ – F_min_)/(F_max_ – F_min_). To normalize the responses, the minimum values during the basal period (F_min_) was subtracted from the responses every 3 s (F_res_) and the maximum value obtained due to addition of the ionomycin (F_max_) (#I0634, Sigma, MA). After the normalization, the maximum increase in Ca^2+^_i_ (Ca^2+^_i_ max) during the stimulation period was extracted for further analysis.

### Statistics

The data are presented as moving averages ± SEMs or means ± SEMs. The number of times each experiment was performed (*N*) is indicated in the figure legends. The normality of the data was assessed using the Shapiro-Wilk test. If the data were normally distributed, Student’s-*t* test or Welch’s-*t* test was conducted for pairwise comparisons, and one-way analysis of variance (ANOVA) with the Dunnett’s or Tukey’s *post hoc* analysis was performed for multiple pairwise comparisons. For non-normally distributed data, the Mann-Whitney *U* test was performed for pairwise comparisons, and non-parametric ANOVA (Kruskal-Wallis test) followed by *post hoc* analysis with the Steel test or Steel-Dwass test was conducted for multiple pairwise comparisons. All statistical analyses were performed using EZR (version 1.61; Saitama Medical Center, Jichi Medical University) (Kanda, 2013), which is a graphical user interface for R (The R Foundation for Statistical Computing). Statistical significance is indicated by asterisks, where *P < 0.05, **P < 0.01, and ***P < 0.001. NS denotes not significant.

## Data availability statement

The original contributions presented in this study are included in this article/Supplementary material, further inquiries can be directed to the corresponding author.

## Ethics statement

The manuscript presents research on animals that do not require ethical approval for their study.

## Author contributions

SS, AMM, and TS contributed to the conception and design of the study. SS and AMM performed the experiments and analysis. SS and TS wrote the first draft of the manuscript. AMM wrote sections of the manuscript. All authors contributed to manuscript revision and read and approved the submitted version.
