# Supplementary material for: Avoidance of thiazoline compound depends on multiple sensory pathways mediated by TrpA1 and ORs in Drosophila
Source: Front Mol Neurosci. 2023 Dec 22;16:1249715. doi: 10.3389/fnmol.2023.1249715 (PMC10771277; doi:10.3389/fnmol.2023.1249715)
Supplement: Supplementary file 1 [file Data_Sheet_1.docx]

Supplementary Material

**Avoidance of thiazoline compound depends on multiple sensory pathways mediated by TrpA1 and ORs in *Drosophila***

**Shoma Sato, Aliyu Mudassir Magaji, Makoto Tominaga, Takaaki Sokabe^*^**

*** Correspondence:** Corresponding Author: sokabe@nips.ac.jp

**Supplementary Figure 1. Schematic of the setup and the analysis for the two-way positional choice preference assay.**

**(A)** Schematic illustrates the two-choice positional preference choice assay. Starved male flies were placed on assay plates coated with sucrose-containing agarose. Each plate was divided into four sections, with two diagonal quadrants containing a test chemical (Chemical) and the other two quadrants serving as controls without the chemical (Control). The assay plates were illuminated with red light from the bottom and still images of assay plates were captured from the top every one minute using a digital camera for a duration of up to 120 minutes. The image on the right side demonstrates avoidance behavior observed in response to sections containing chemical. **(B)** An example of individual traces depicting the temporal changes of preference index (PI) to 1 mM 2MT in control flies (*N* = 12, same data shown in Figure 2A). Left: raw data, where the PI at each minute is plotted for each sample. Right: the PIs calculated as 21-minute moving averages. The mean ± SEM values obtained from these traces were calculated and presented in all the figures.

**Supplementary Figure 2. 2MT evoked avoidance behavior in adult flies** **(related to Figure 1)**

The PI to chemicals at 30 and 90 minutes. **(A)** The PI of 24-hour-starved (black) or non-starved (red) flies for 100 μM quinine. The PI of 24-hour-starved flies for 100 μM quinine in the absence of sucrose (blue). The PI of 24-hour-starved flies in the absence of quinine (green). The same letters indicate no significant difference as determined by one-way ANOVA with Tukey’s multiple comparison or Kruskal-Wallis test with Steel-Dwass multiple comparison (*N* = 8). **(B)** The PI of 24-hour-starved (black) or non-starved (red) flies for 1 mM 2MT. The PI of 24-hour-starved flies for 1 mM 2MT in the absence of sucrose (blue). NS, not significant; **P* < 0.05; ****P* < 0.001 by one-way ANOVA with Tukey’s multiple comparison or Kruskal-Wallis test with Steel-Dwass multiple comparison (*N* = 9).

**Supplementary Figure 3. 2MT avoidance was reduced in *TrpA1* or *Orco* mutants (related to Figure 2).**

The PI to 2MT at 30 and 90 minutes. **(A)** The PI to 1 mM 2MT in control (*w^1118^*; black), *TrpA1^1^* (red), and *Orco^2^* (blue) flies. ***P* < 0.01; ****P* < 0.001 by one-way ANOVA with Tukey’s multiple comparison or Kruskal-Wallis test with Steel-Dwass multiple comparison (*N* = 9–12). **(B)** The PI to 1 mM 2MT in control (*w^1118^*; black), *pain^4^* (red), *wtrw^2^* (blue) flies. NS, not significant; **P* < 0.05; ****P* < 0.001 by one-way ANOVA with Dunnett’s multiple comparison or Kruskal-Wallis test with Steel multiple comparison (*N* = 8). **(C)** The PI to 1 mM 2MT in control (*w^1118^*; black), *Trp^MB03672^* (*Trp^MB^*; red), *Trpl^MB10553^* (*Trpl^MB^*; blue), *Trpγ^G4^* (green) flies. NS, not significant by one-way ANOVA with Dunnett’s multiple comparison or Kruskal-Wallis test with Steel multiple comparison (*N* = 7–8). **(D)** The PI to 100 μM 2MT in control (*w^1118^*; black), *TrpA1^1^* (red), and *Orco^2^* (blue) flies. NS, not significant; **P* < 0.05; ***P* < 0.01 by Kruskal-Wallis test with Steel-Dwass multiple comparison (*N* = 9).

**Supplementary Figure 4. 2MT avoidance was reduced in *TrpA1* or *Orco* mutants (related to Figure 2).**

The temporal changes of PI to different doses of 2MT **(A–F)** and the PI at 30, 60, and 90 minutes **(G)**. Dotted lines in **A–F** represent the level indicating no preference or avoidance. The data are presented as moving average ± SEM. The PI to 10 mM (**A**), 3 mM (**B**), 600 μM (**C**), 300 μM (**D**), 30 μM (**E**), and 10 μM (**F**) 2MT in control (*w^1118^*; black), *TrpA1^1^* (red), and *Orco^2^* (blue) flies. NS, not significant; **P* < 0.05; ***P* < 0.01; ****P* < 0.001 by one-way ANOVA with Tukey’s multiple comparison or Kruskal-Wallis test with Steel-Dwass multiple comparison (*N* = 6–12).

**Supplementary Figure 5. Locomotor activities of flies in the presence of 2MT (related to Figure 2).**

The total distance moved in control (*w^1118^*; **A**) and *TrpA1^1^* flies (**B**) during 10-minutes test intervals at 0, 30, and 60 minutes after the start of the recording. Flies were exposed to 100 μM (yellow), 1 mM (orange),10 mM (red) 2MT, and in the absence of 2MT (white). The data are presented as mean ± SEM. NS, not significant; ***P* < 0.01; ****P* < 0.001 by one-way ANOVA with Dunnett’s multiple comparison or Kruskal-Wallis test with Steel multiple comparison (*N* = 36).

**Supplementary Figure 6. Thiazoline-related compounds evoked different behavioral responses (related to Figure 3).**

The PI to thiazoline-related compounds at 30 and 90 minutes. **(A)** The PI to 300 μM 4E2MT in control (*w^1118^*; black), *TrpA1^1^* (red), and *Orco^2^* (blue) flies. NS, not significant; **P* < 0.05; ***P* < 0.01 by one-way ANOVA with Tukey’s multiple comparison (*N* = 9). **(B)** The PI to 100 μM TMO in control (*w^1118^*; black) and *TrpA1^1^* (red) flies. NS, not significant by Student’s *t*-test (*N* = 10). **(C)** The PI to 1 mM 2MO in control (*w^1118^*; black) and *TrpA1^1^* (red) flies. NS, not significant by Student’s *t*-test (*N* = 8).

**Supplementary Figure 7. TrpA1 expression in taste and nociceptive sensory neurons was required for 2MT avoidance (related to Figure 4).**

The PI to 2MT at 30 and 90 minutes. **(A)** The PI to 1 mM 2MT in *Gr66a-GAL4*/+ (black), +/*UAS*-*dicer2*; +/*UAS*-*TrpA1* RNAi (*TrpA1* RNAi/+; blue), and *Gr66a-GAL4*/*UAS*-*dicer2*; +/*UAS*-*TrpA1* RNAi (*Gr66a* > *TrpA1* RNAi; red) flies (left). The PI to 1 mM 2MT in *ppk-GAL4*/+ (black), *TrpA1* RNAi/+ (blue), and +/*UAS*-*dicer2*; *ppk-GAL4*/*UAS*-*TrpA1* RNAi (*ppk* > *TrpA1* RNAi; red) flies (middle). The PI to 1 mM 2MT in *Orco-GAL4*/+ (black), *TrpA1* RNAi/+ (blue), and *Orco-GAL4*/*UAS*-*dicer2*; +/*UAS*-*TrpA1* RNAi (*Orco* > *TrpA1* RNAi; red) flies (right). NS, not significant; **P* < 0.05; ***P* < 0.01 by Kruskal Wallis test with Steel-Dwass multiple comparison (*N* = 8; note that *TrpA1* RNAi/+ data are shared between left and middle). **(B)** The PI to 100 μM 2MT (left) and 1 mM 2MT (right) in *Orco*-*GAL4*/+ (black), +/*UAS*-*Kir2.1::GFP* (*Kir2.1*/+; blue), and *Orco*-*GAL4*/*UAS*-*Kir2.1::GFP* (*Orco* > *Kir2.1*; red) flies. NS, not significant; *P < 0.05; **P < 0.01 by Kruskal-Wallis test with Steel-Dwass multiple comparison (*N* = 9–10).

**Supplementary Figure 8. TrpA1-C and TrpA1-D isoform were involved in 2MT avoidance (related to Figure 5).**

The PI to 1 mM 2MT at 30 and 90 minutes. **(A)** The PI to 1 mM 2MT in *TrpA1^1^*/*TrpA1-T2A-GAL4* (*TrpA1-KI*; black), *TrpA1^1^*/*TrpA1-KO* (*TrpA1-KO*; green), *TrpA1^1^/TrpA1-CKI-T2A-GAL4* (*TrpA1-CKI*; blue), and *TrpA1^1^*/*TrpA1-DKI-T2A-GAL4* (*TrpA1-DKI*; red) flies. The same letters indicate no significant difference by one-way ANOVA with Tukey’s multiple comparison (*N* = 12–16). **(B)** The PI to 1 mM 2MT in *TrpA1-KI* (black), *TrpA1-KO* (green), *TrpA1^1^*/*TrpA1-AKI-T2A-GAL4* (*TrpA1-AKI*; blue), *TrpA1^1^*/*TrpA1-BKI-T2A-GAL4* (*TrpA1-BKI*; red), and *TrpA1^1^*/*TrpA1-EKI-T2A-GAL4* (*TrpA1-EKI*; purple) flies. The same letters indicate no significant difference by Kruskal-Wallis test with Steel-Dwass multiple comparison (*N* = 12–21).

**Supplementary Figure 9. Expression patterns of TrpA1-A and TrpA1-B isoforms in the legs (related to Figure 6).**

The GFP expression in the forelegs (top), the midlegs (middle), and the hindlegs (bottom). Arrows indicate cell bodies of *ppk*-neurons located in the 1st–3rd segments of tarsus. Arrowheads indicate cell bodies of *ppk*/*Gr66a*-neurons located in the 5th segment of protarsus in the forelegs. Magnified images of cell bodies are shown on the right. **(A)** *TrpA1-T2A-GAL4*/*UAS*-*mCD8::GFP* merged with brightfield images (same samples shown in Figure 6A). **(B)** *TrpA1-AKI-T2A-GAL4*/*UAS*-*mCD8::GFP*. **(C)** *TrpA1-BKI-T2A-GAL4*/*UAS*-*mCD8::GFP*. Scale bars represent 100 μm (left) or 10 μm (right). The expression patterns were confirmed in three individuals.

**Supplementary Figure 10. Expression patterns of TrpA1 in the labellum (related to Figure 6).**

The mCherry (left), GFP (middle), and merged (right) images in the labellum. **(A)** *lexAop2*-*mCherry*/*Gr66a-GAL4*; *TrpA1-T2A-LexA*/*UAS*-*mCD8::GFP*. Arrowheads exemplify cell bodies exclusively expressing *TrpA1*. **(B)** *lexAop2*-*mCherry*/*UAS*-*mCD8::GFP*; *TrpA1-T2A-LexA*/*ppk-GAL4*. Scale bars represent 100 μm. The expression patterns were confirmed in three individuals.

**Supplementary Figure 11. Comparison of TrpA1 sequence between mice and *Drosophila* (related to Figure 7).**

An alignment of amino acid sequences of mouse TRPA1 (mTRPA1), *Drosophila* TrpA1-C (dTrpA1-C), and *Drosophila* TrpA1-D (dTrpA1-D). Green and blue letters indicate the alternatively spliced exons present in TrpA1-C and TrpA1-D, respectively (see green and blue boxes in **Figure 7G**). The five cysteine residues required for 2MT response in mouse TRPA1 are indicated by arrowheads (C415, C422, C666, C714, and C859). Additional cysteine (C66) required for 2MT response in mice is not shown. Gray boxes are conserved cysteines in dTrpA1-C (C480) and dTrpA1-D (C480 and C729).

**Supplementary Figure 12. Citronellal activated TrpA1-C, TrpA1-D, and TrpA1-D double Cys mutant (related to Figure 7).**

The quantification of the maximal Ca^2+^_i_ increase (Ca^2+^_i_ max) in response to 1 mM citronellal in cells expressing TrpA1-C (red), TrpA1-D (blue), and TrpA1-D C480S/C729S (purple). The data are presented as mean ± SEM (*N* = 6–7).

**Supplementary Figure 13. Conservation of cysteine in TrpA1 among insect species.**

An alignment of amino acid sequences of insect TrpA1. The two corresponding cysteine residues react with 2MT in *Drosophila melanogaster* TrpA1-D (*D. melanogaster*) are indicated by arrowheads (C480 and C729). *D*. *suzukii*: *Drosophila* *suzukii* (XP_036671523.1); *B*. *germanica*: *Blattella* *germanica* (PSN57930.1); *S*. *furcifera*: *Sogatella* *furcifera* (QMS80393.1); *N*. *lugens*: *Nilaparvata* *lugens* (AOR81469.1); *C*. *suppressalis*: *Chilo* *suppressalis* (QLD94937.1); *B*. *tabaci*: *Bemisia* *tabaci* (WMY99264.1); *L*. *hesperus*: *Lygus* *hesperus* (QIS92841.1). Gray boxes indicate conserved cysteines.

TrpA1-C cDNA sequence

ATGCCCAAGCTCTACAACGGAGTCTACAGCGGTCAGTGCGGCGCCCTATCGCCACCTGACCTCATGGAGGCCCAGCCGAAGCTACTTCCCAAGCCAAGGAGCAACAGCAGCGGCAGCACCGGCCGGAACAGCAAGTATTGGATATTTTCAATGATAATCGAGCGCAGTGCGGGTCCCAAGCGGATTGAAATCGATGGCGATGATGCGGACACGCCGCTGGAGGCCATCCTGCCAGCCGAACCGCCGGCGGAGGTCTGCCTCTTGCGTGACAGCCCCTTCAGGATATTGCGGGCGGCTGAGTCCGGAAACCTTGACGACTTCAAGCGACTCTTCATGGCGGACAACTCGCGCATTGCTTTAAAGGATGCGAAAGGACGAACGGCTGCCCATCAGGCGGCGGCCCGTAATAGGGTTAACATTTTGCGGTACATTCGCGACCAGAATGGCGACTTTAATGCGAAGGATAATGCCGGCAATACCCCGCTCCACATCGCCGTGGAGAGCGATGCCTACGACGCTCTGGACTATCTATTGTCCATCCCAGTGGATACGGGAGTGCTGAACGAGAAGAAGCAGGCACCAGTGCACTTGGCCACCGAGCTGAACAAAGTGAAGTCCCTTCGGGTGATGGGTCAGTACCGCAATGTCATCGATATTCAGCAGGGCGGCGAACATGGACGTACCGCTCTGCACTTGGCCGCCATCTATGATCACGAGGAGTGCGCTCGCATCCTGATAACTGAGTTCGATGCATGCCCACGTAAGCCCTGTAACAATGGTTATTATCCCATACACGAAGCGGCCAAGAATGCCAGCTCCAAGACAATGGAGGTCTTCTTCCAGTGGGGCGAGCAGCGCGGCTGCACCCGCGAGGAGATGATATCCTTCTACGACTCGGAGGGCAATGTGCCGCTCCATTCGGCTGTCCATGGTGGCGACATCAAGGCTGTGGAGCTGTGCCTCAAGTCCGGGGCCAAGATATCTACGCAGCAACACGATCTCTCGACGCCAGTGCACCTGGCTTGTGCCCAGGGAGCCATAGACATTGTGAAGCTCATGTTCGAGATGCAGCCAATGGAGAAGCGACTATGTCTTAGTTGCACGGATGTGCAGAAGATGACGCCGCTGCACTGCGCCTCCATGTTCGATCATCCGGACATTGTGTCCTATCTGGTAGCCGAGGGAGCGGACATCAATGCCCTGGACAAGGAGCATCGCTCTCCGTTGCTCTTGGCGGCATCGCGTAGCGGTTGGAAAACGGTCCACCTCCTGATTCGCCTGGGGGCGTGCATTAGTGTGAAGGACGCCGCCGCCCGCAATGTGCTGCACTTCGTCATCATGAACGGCGGCCGGCTGACGGACTTCGCGGAGCAGGTGGCCAACTGCCAGACGCAGGCGCAACTGAAGCTGCTGCTCAACGAGAAGGACAGCATGGGCTGCTCACCGCTGCACTACGCCAGTCGGGATGGGCACATCCGTTCGTTGGAGAACCTCATTCGACTGGGAGCCTGCATCAACCTGAAGAACAACAACAACGAGAGTCCGCTGCACTTTGCCGCTCGTTACGGAAGATACAATACGGTGCGGCAGCTCTTGGACTCCGAGAAGGGATCCTTCATCATCAACGAAAGTGACGGTGCAGGGATGACACCTCTGCACATATCCTCGCAGCAAGGACACACGCGAGTGGTGCAGCTGCTACTCAATCGAGGAGCCCTGCTCCATCGGGACCACACCGGACGCAATCCTCTCCAGCTAGCGGCCATGTCCGGATATACCGAGACCATCGAGCTGCTGCACTCGGTGCACTCGCATCTGCTCGATCAGGTGGATAAGGATGGGAACACCGCTCTTCACCTGGCCACCATGGAGAATAAGCCCCATGCGATCTCCGTGCTGATGTCTATGGGCTGTAAGCTGGTCTACAACGTTCTGGACATGAGTGCCATTGACTATGCCATCTACTACAAATATCCGGAGGCTGCCCTGGCCATGGTCACCCACGAGGAGCGGGCCAACGAGGTGATGGCTCTGCGTTCCGACAAGCATCCGTGCGTGACCCTCGCCCTAATTGCCTCCATGCCCAAGGTATTCGAGGCGGTGCAGGACAAGTGCATTACCAAGGCCAATTGCAAGAAGGACTCGAAGAGTTTCTACATAAAATATTCGTTTTGGCCCTACCAAAAGACACCCGAACAGATTGAGGCCAAGCGCAAAGAGTTCAATGACCCCAAGTGGCGACCCGCGCCTTTGGCCGTGGTGAACACCATGGTAACACATGGCAGGGTGGAGCTGCTGGCCCATCCGCTCAGTCAGAAGTATCTGCAGATGAAGTGGAACTCCTACGGCAAGTACTTTCACCTGGCCAACCTGCTAATCTACTCGATATTCCTGGTCTTTGTAACCATCTACTCTTCGCTGATGATGAACAACATCGAACTGAAGGCTGGGGACAACAAGACGATGAGTCAATACTGCAATATGGGATGGGAGCAGCTGACCATGAATCTCTCGCAGAACCCGTCGGTGGCATCACAGATTCGTTTGGATTCCTGCGAGGAGCGTATAAATAGAACCACTGCAATACTTTTCTGTGCGGTGGTCATCGTGGTCTATATACTGCTCAACTCGATGCGGGAACTAATACAGATATACCAGCAGAAATTGCACTATATCCTGGAGACAGTTAATTTGATATCCTGGGTGCTGTACATCTCGGCTTTGGTGATGGTAACACCGGCATTTCAGCCGGATGGAGGAATCAATACCATTCATTACTCGGCCGCTTCAATAGCAGTCTTTCTGTCGTGGTTCCGATTGCTACTGTTCCTGCAAAGATTCGACCAGGTCGGCATCTATGTGGTCATGTTCTTGGAGATTCTGCAGACGCTCATTAAAGTGCTGATGGTATTCTCCATACTTATAATCGCCTTTGGTCTGGCTTTCTATATACTACTTTCAAAGATTATTGACCCCCAACCGAACCACTTGTCCTTCTCCAACATACCCATGTCCTTGCTGCGAACTTTCTCAATGATGCTGGGCGAGCTGGACTTTGTGGGTACCTATGTGAACACCTACTATCGGGATCAGTTGAAGGTGCCCATGACATCCTTTTTGATTTTGAGTGTCTTTATGATCCTTATGCCCATTCTTCTGATGAACTTGCTCATCGGTTTGGCCGTCGGCGATATTGAGTCAGTGCGTCGCAATGCCCAGCTCAAGAGACTGGCCATGCAGGTGGTGCTCCACACGGAGCTGGAGAGGAAGTTGCCCCATGTCTGGCTGCAGCGAGTTGACAAGATGGAGCTGATTGAGTATCCCAATGAAACCAAGTGCAAGCTGGGCTTCTGCGATTTCATCCTGCGCAAGTGGTTCTCGAATCCATTCACCGAGGATTCCTCCATGGACGTCATCTCCTTCGACAACAATGATGACTACATCAACGCAGAATTGGAACGGCAGAGGCGAAAGTTGCGCGACATAAGTCGCATGCTGGAGCAACAGCACCATCTGGTTCGGCTTATTGTCCAAAAGATGGAGATCAAGACGGAGGCGGATGACGTGGACGAGGGTATATCCCCAAACGAGTTGCGATCCGTCGTCGGTTTGAGATCGGCAGGCGGAAATCGATGGAACTCGCCGCGAGTCCGGAATAAACTCCGAGCCGCCCTGAGCTTCAATAAGAGCATGTAG

TrpA1-D cDNA sequence

ATGCCCAAGCTCTACAACGGAGTCTACAGCGGTCAGTGCGGCGCCCTATCGCCACCTGACCTCATGGAGGCCCAGCCGAAGCTACTTCCCAAGCCAAGGAGCAACAGCAGCGGCAGCACCGGCCGGAACAGCAAGTATTGGATATTTTCAATGATAATCGAGCGCAGTGCGGGTCCCAAGCGGATTGAAATCGATGGCGATGATGCGGACACGCCGCTGGAGGCCATCCTGCCAGCCGAACCGCCGGCGGAGGTCTGCCTCTTGCGTGACAGCCCCTTCAGGATATTGCGGGCGGCTGAGTCCGGAAACCTTGACGACTTCAAGCGACTCTTCATGGCGGACAACTCGCGCATTGCTTTAAAGGATGCGAAAGGACGAACGGCTGCCCATCAGGCGGCGGCCCGTAATAGGGTTAACATTTTGCGGTACATTCGCGACCAGAATGGCGACTTTAATGCGAAGGATAATGCCGGCAATACCCCGCTCCACATCGCCGTGGAGAGCGATGCCTACGACGCTCTGGACTATCTATTGTCCATCCCAGTGGATACGGGAGTGCTGAACGAGAAGAAGCAGGCACCAGTGCACTTGGCCACCGAGCTGAACAAAGTGAAGTCCCTTCGGGTGATGGGTCAGTACCGCAATGTCATCGATATTCAGCAGGGCGGCGAACATGGACGTACCGCTCTGCACTTGGCCGCCATCTATGATCACGAGGAGTGCGCTCGCATCCTGATAACTGAGTTCGATGCATGCCCACGTAAGCCCTGTAACAATGGTTATTATCCCATACACGAAGCGGCCAAGAATGCCAGCTCCAAGACAATGGAGGTCTTCTTCCAGTGGGGCGAGCAGCGCGGCTGCACCCGCGAGGAGATGATATCCTTCTACGACTCGGAGGGCAATGTGCCGCTCCATTCGGCTGTCCATGGTGGCGACATCAAGGCTGTGGAGCTGTGCCTCAAGTCCGGGGCCAAGATATCTACGCAGCAACACGATCTCTCGACGCCAGTGCACCTGGCTTGTGCCCAGGGAGCCATAGACATTGTGAAGCTCATGTTCGAGATGCAGCCAATGGAGAAGCGACTATGTCTTAGTTGCACGGATGTGCAGAAGATGACGCCGCTGCACTGCGCCTCCATGTTCGATCATCCGGACATTGTGTCCTATCTGGTAGCCGAGGGAGCGGACATCAATGCCCTGGACAAGGAGCATCGCTCTCCGTTGCTCTTGGCGGCATCGCGTAGCGGTTGGAAAACGGTCCACCTCCTGATTCGCCTGGGGGCGTGCATTAGTGTGAAGGACGCCGCCGCCCGCAATGTGCTGCACTTCGTCATCATGAACGGCGGCCGGCTGACGGACTTCGCGGAGCAGGTGGCCAACTGCCAGACGCAGGCGCAACTGAAGCTGCTGCTCAACGAGAAGGACAGCATGGGCTGCTCACCGCTGCACTACGCCAGTCGGGATGGGCACATCCGTTCGTTGGAGAACCTCATTCGACTGGGAGCCTGCATCAACCTGAAGAACAACAACAACGAGAGTCCGCTGCACTTTGCCGCTCGTACGGAAGATACAATACGGTGCGGCAGCTCTTGGATCTCCGAGAAGGGATCCTTCATCATCAACGAAAGTGACGGTGCAGGGATGACACCTCTGCACATATCCTCGCAGCAAGGACACACGCGAGTGGTGCAGCTGCTACTCAATCGAGGAGCCCTGCTCCATCGGGACCACACCGGACGCAATCCTCTCCAGCTAGCGGCCATGTCCGGATATACCGAGACCATCGAGCTGCTGCACTCGGTGCACTCGCATCTGCTCGATCAGGTGGATAAGGATGGGAACACCGCTCTTCACCTGGCCACCATGGAGAATAAGCCCCATGCGATCTCCGTGCTGATGTCTATGGGCTGTAAGCTGGTCTACAACGTTCTGGACATGAGTGCCATTGACTATGCCATCTACTACAAATATCCGGAGGCTGCCCTGGCCATGGTCACCCACGAGGAGCGGGCCAACGAGGTGATGGCTCTGCGTTCCGACAAGCATCCGTGCGTGACCCTCGCCCTAATTGCCTCCATGCCCAAGGTATTCGAGGCGGTGCAGGACAAGTGCATTACCAAGGCCAATTGCAAGAAGGACTCGAAGAGTTTCTACATCAAATACTCTTTCGCATTCTTGCAATGCCCCTTTATGTTTGCCAAGATTGATGAGAAAACCGGAGAGTCGATTACGACCGCCAGTCCCATTCCGTTGCCGGCTTTGAATACCATGGTAACACATGGCAGGGTGGAGCTGCTGGCCCATCCGCTCAGTCAGAAGTATCTGCAGATGAAGTGGAACTCCTACGGCAAGTACTTTCACCTGGCCAACCTGCTAATCTACTCGATATTCCTGGTCTTTGTAACCATCTACTCTTCGCTGATGATGAACAACATCGAACTGAAGGCTGGGGACAACAAGACGATGAGTCAATACTGCAATATGGGATGGGAGCAGCTGACCATGAATCTCTCGCAGAACCCGTCGGTGGCATCACAGATTCGTTTGGATTCCTGCGAGGAGCGTATAAATAGAACCACTGCAATACTTTTCTGTGCGGTGGTCATCGTGGTCTATATACTGCTCAACTCGATGCGGGAACTAATACAGATATACCAGCAGAAATTGCACTATATCCTGGAGACAGTTAATTTGATATCCTGGGTGCTGTACATCTCGGCTTTGGTGATGGTAACACCGGCATTTCAGCCGGATGGAGGAATCAATACCATTCATTACTCGGCCGCTTCAATAGCAGTCTTTCTGTCGTGGTTCCGATTGCTACTGTTCCTGCAAAGATTCGACCAGGTCGGCATCTATGTGGTCATGTTCTTGGAGATTCTGCAGACGCTCATTAAAGTGCTGATGGTATTCTCCATACTTATAATCGCCTTTGGTCTGGCTTTCTATATACTACTTTCAAAGATTATTGACCCCCAACCGAACCACTTGTCCTTCTCCAACATACCCATGTCCTTGCTGCGAACTTTCTCAATGATGCTGGGCGAGCTGGACTTTGTGGGTACCTATGTGAACACCTACTATCGGGATCAGTTGAAGGTGCCCATGACATCCTTTTTGATTTTGAGTGTCTTTATGATCCTTATGCCCATTCTTCTGATGAACTTGCTCATCGGTTTGGCCGTCGGCGATATTGAGTCAGTGCGTCGCAATGCCCAGCTCAAGAGACTGGCCATGCAGGTGGTGCTCCACACGGAGCTGGAGAGGAAGTTGCCCCATGTCTGGCTGCAGCGAGTTGACAAGATGGAGCTGATTGAGTATCCCAATGAAACCAAGTGCAAGCTGGGCTTCTGCGATTTCATCCTGCGCAAGTGGTTCTCGAATCCATTCACCGAGGATTCCTCCATGGACGTCATCTCCTTCGACAACAATGATGACTACATCAACGCAGAATTGGAACGGCAGAGGCGAAAGTTGCGCGACATAAGTCGCATGCTGGAGCAACAGCACCATCTGGTTCGGCTTATTGTCCAAAAGATGGAGATCAAGACGGAGGCGGATGACGTGGACGAGGGTATATCCCCAAACGAGTTGCGATCCGTCGTCGGTTTGAGATCGGCAGGCGGAAATCGATGGAACTCGCCGCGAGTCCGGAATAAACTCCGAGCCGCCCTGAGCTTCAATAAGAGCATGTAG

**Supplementary Figure 14. Entire sequence of the coding region of *Drosophila* TrpA1-C and TrpA1-D.**

The cDNA sequences of the cloned *TrpA1-C* and *TrpA1-D*. Red bases indicate nucleotide mismatches compared with the reference sequence in the database (C isoform vs. NM_001274672_H; D isoform vs. NM_001274672_G). The amino acid sequence of both isoforms matched completely with those in the database.

**Supplementary Table 1.** **PCR primers designed for the cloning of *TrpA1-C* and *TrpA1-D***

| Forward (5’ - 3’) | Reverse (5’ - 3’) |
| --- | --- |
| AGCGGCCGCCACCATGCCCAAGCTCTACAACG | ATCTAGACTACATGCTCTTATTGAAGCTCAGG |

*Red letters indicate KOZAC sequence.

**Supplementary Table 2. PCR primers designed for the site-directed mutagenesis in *TrpA1-C* and *TrpA1-D***

| Mutation | Forward (5’ - 3’) | Reverse (5’ - 3’) |
| --- | --- | --- |
| C480S | GACAGCATGGGCAGCTCGCCGCTGC | GCAGCGGCGAGCTGCCCATGCTGTC |
| C729S | TTCGCATTCTTGCAAAGCCCCTTTATGTTTGC | AGGGGCTTTGCAAGAATGCGAAAGAGTATTTG |

*Red letters denote codons for serine, which replace the original cysteine codon.
